# MAMILNet: advancing precision oncology with multi-scale attentional multi-instance learning for whole slide image analysis

**DOI:** 10.3389/fonc.2024.1275769

**Published:** 2024-04-30

**Authors:** Qinqing Wang, Qiu Bi, Linhao Qu, Yuchen Deng, Xianhong Wang, Yijun Zheng, Chenrong Li, Qingyin Meng, Kun Miao

**Affiliations:** ^1^ Department of Pathology, The First People’s Hospital of Yunnan Province, The Affiliated Hospital of Kunming University of Science and Technology, Kunming, Yunnan, China; ^2^ Department of MRI, The First People’s Hospital of Yunnan Province, The Affiliated Hospital of Kunming University of Science and Technology, Kunming, Yunnan, China; ^3^ Fudan University, Shanghai, China; ^4^ School of Clinical Medicine, The Affiliated Hospital of Kunming University of Science and Technology, Kunming, Yunnan, China; ^5^ Department of Pathology, The Third Affiliated Hospital of Kunming Medical University (Tumor Hospital of Yunnan Province), Kunming, Yunnan, China; ^6^ Department of Medical Oncology, The First People’s Hospital of Yunnan Province, The Affiliated Hospital of Kunming University of Science and Technology, Kunming, Yunnan, China

**Keywords:** whole slide image analysis, multiple instance learning, cancer diagnosis, multi-scale attention, deep learning

## Abstract

**Background:**

Whole Slide Image (WSI) analysis, driven by deep learning algorithms, has the potential to revolutionize tumor detection, classification, and treatment response prediction. However, challenges persist, such as limited model generalizability across various cancer types, the labor-intensive nature of patch-level annotation, and the necessity of integrating multi-magnification information to attain a comprehensive understanding of pathological patterns.

**Methods:**

In response to these challenges, we introduce MAMILNet, an innovative multi-scale attentional multi-instance learning framework for WSI analysis. The incorporation of attention mechanisms into MAMILNet contributes to its exceptional generalizability across diverse cancer types and prediction tasks. This model considers whole slides as “bags” and individual patches as “instances.” By adopting this approach, MAMILNet effectively eliminates the requirement for intricate patch-level labeling, significantly reducing the manual workload for pathologists. To enhance prediction accuracy, the model employs a multi-scale “consultation” strategy, facilitating the aggregation of test outcomes from various magnifications.

**Results:**

Our assessment of MAMILNet encompasses 1171 cases encompassing a wide range of cancer types, showcasing its effectiveness in predicting complex tasks. Remarkably, MAMILNet achieved impressive results in distinct domains: for breast cancer tumor detection, the Area Under the Curve (AUC) was 0.8872, with an Accuracy of 0.8760. In the realm of lung cancer typing diagnosis, it achieved an AUC of 0.9551 and an Accuracy of 0.9095. Furthermore, in predicting drug therapy responses for ovarian cancer, MAMILNet achieved an AUC of 0.7358 and an Accuracy of 0.7341.

**Conclusion:**

The outcomes of this study underscore the potential of MAMILNet in driving the advancement of precision medicine and individualized treatment planning within the field of oncology. By effectively addressing challenges related to model generalization, annotation workload, and multi-magnification integration, MAMILNet shows promise in enhancing healthcare outcomes for cancer patients. The framework’s success in accurately detecting breast tumors, diagnosing lung cancer types, and predicting ovarian cancer therapy responses highlights its significant contribution to the field and paves the way for improved patient care.

## 1 Introduction

In recent years, computational pathology has emerged as a transformative discipline with immense potential to revolutionize cancer diagnosis and treatment planning. The advent of digital pathology and whole slide imaging has led to vast histopathological data repositories, presenting an unprecedented opportunity for deep learning networks in this field Srinidhi et al. (1) Qu et al. (2). Whole Slide Image (WSI) analysis, enabled by deep learning algorithms, shows promise in tumor detection, typing, and drug treatment response prediction, heralding a new era of precision medicine in oncology Cheplygina et al. (3) Rony et al. (4).

Tumor detection is critical for timely and accurate cancer diagnoses. Conventional methods, relying on manual examination by pathologists, can be time-consuming and subjective, leading to diagnostic errors and variability. Deep learning networks revolutionize tumor detection, using Convolutional Neural Networks (CNNs) to meticulously analyze digitized histopathological images, identifying malignancy with precision and efficiency. Integrating deep learning expedites diagnostic processes, enhances accuracy, and ensures reproducibility in clinical settings. Tumor typing, categorizing cancers into subtypes, is vital for personalized oncology. Deep learning networks address challenges in tumor typing, comprehensively learning from annotated histopathological datasets. They discern subtle differences between tumor subtypes with remarkable accuracy, facilitating efficient and precise tumor typing, leading to tailored therapies and improved patient outcomes. In the realm of cancer treatment, establishing deep-learning models to predict drug therapy response from WSIs has emerged as a transformative frontier. Traditional methods relying on manual evaluation of biopsy samples struggle to capture the true heterogeneity of tumor responses. In contrast, deep learning networks in WSI analysis offer a promising and powerful approach. By analyzing large-scale histopathological datasets, these models can detect subtle alterations induced by therapies, enabling accurate and timely prediction of treatment response. This groundbreaking development holds the potential to revolutionize cancer treatment and elevate patient outcomes to new heights.

Deep learning networks have a significant impact on computational pathology, particularly in WSI analysis for tumor detection, typing, and treatment response prediction, advancing precision medicine and patient care. However, integrating deep learning algorithms faces significant challenges in this domain. A primary issue is the limited generalizability of models across different cancer types and tasks, compromising their practicality for routine clinical use. Many current approaches achieve remarkable results on specific cancer types or tasks, but their performance tends to deteriorate when confronted with diverse cancers. The burden of patch-level annotation is another major challenge. WSIs are massive and need to be divided into smaller patches for deep learning training. Manual annotation of these patches is time-consuming and labor-intensive, making comprehensive annotation impractical, hindering the adoption of fully-supervised algorithms. Moreover, handling multi-magnification information is vital. Current studies often focus on single magnification analysis, neglecting the diagnostic information embedded in multiple magnifications. This limitation may lead to incomplete understanding of pathological patterns, reducing the efficacy of deep learning algorithms in capturing the full complexity of the images Srinidhi et al. (1) Qu et al. (2) Cheplygina et al. (3) Rony et al. (4) Wang et al. (5) Qu et al. (6).

This paper presents MAMILNet, a novel multi-scale attentional multi-instance learning framework for whole slide pathological image processing. MAMILNet offers several key advantages over existing methods. Firstly, it exhibits high generalization across multiple cancer types and prediction tasks by skillfully integrating the attention mechanism. This adaptability ensures robust performance in diverse scenarios. Secondly, MAMILNet employs a multi-instance learning (MIL) architecture, treating slides as “bags” and their cut patches as “instances,” effectively representing slides as a whole. This eliminates the need for fine-grained patch-level labeling, reducing the burden of manual labeling for pathologists. Additionally, MAMILNet utilizes a multi-scale “consultation” training and prediction strategy, training with multiple magnifications and aggregating test results from different scales using a probability ensemble method. This approach significantly enhances prediction accuracy by considering multiple magnifications during diagnosis and prediction. Overall, MAMILNet is a promising framework for achieving high-accuracy predictions in WSI analysis with weak labeling at the slide level.

We present a comprehensive evaluation of MAMILNet’s performance. We focus on three critical diagnosis tasks, involving different cancer types, from four distinct medical centers. These tasks include automatic recognition of sentinel lymph node cancer in breast cancer, automatic typing of lung adenocarcinoma and lung squamous cell carcinoma, and drug resistance diagnosis of high-grade serous ovarian cancer. Through a meticulous analysis of 1711 patients and WSIs, MAMILNet demonstrates remarkable accuracy in predicting these complex tasks. The successful outcomes achieved by MAMILNet in this diverse dataset hold significant implications for cancer diagnosis and personalized treatment planning. These findings further reinforce the potential of deep learning networks in advancing WSI processing, paving the way for improved healthcare outcomes in oncology.

## 2 Related work

### 2.1 Deep-learning-based WSI analysis

Numerous noteworthy studies have been dedicated to addressing significant clinical challenges in the WSI analysis field. For instance, Coudray et al. (7) developed deep-learning models capable of accurately predicting cancer subtypes and genetic mutations, sparking the entire field. Naik et al. (8) presented a deep-learning framework for directly predicting estrogen receptor status from H&E slides. Another notable clinical endeavor was undertaken by Tomita et al. (9), who proposed a grid-based network for performing 4-class classification of high-resolution endoscopic esophagus and gastroesophageal junction mucosal biopsy images from 379 patients. Skrede et al. (10) developed a deep model to analyze conventional H&E-stained slides and effectively predict the prognosis of patients after colorectal cancer surgery. Similarly, in a gastrointestinal tract oncology study, Kather et al. (11) employed a deep model to predict microsatellite instability (MSI) directly from H&E-stained slides. Currently, deep-learning models for WSI analysis have been applied across a wide range of cancer types, including breast, colorectal, lung, liver, cervical, thyroid, and bladder cancers Coudray et al. (7) Bejnordi et al. (12) Chaudhary et al. (13) Campanella et al. (14) Saillard et al. (15) Woerl et al. (16) Anand et al. (17) Velmahos et al. (18) Wessels et al. (19) Li et al. (20) Yang et al. (21).

In contrast to the majority of studies that have focused on specific tasks for individual cancers, our proposed MAMILNet takes a broader approach, exploring network architectures for multiple tasks across multiple cancer species. With MAMILNet, we have successfully achieved high accuracy in predicting multiple tasks for various cancer types.

### 2.2 Multi-instance learning techniques

As an effective weakly supervised learning algorithm, multi-instance learning has emerged as the mainstream method for WSI analysis based on deep learning Campanella et al. (14)Ilse et al. (22)Shi et al. (23) Li et al. (24) Qu et al. (25) Qu et al. (26). Due to the substantial size of WSIs, often reaching 100,000 × 100,000 pixels, direct utilization as input for deep-learning models is impractical. To alleviate the computational burden, WSIs are typically divided into numerous small patches for processing. In multi-instance learning, each WSI is treated as a “bag,” while the segmented patches are regarded as “instances” belonging to that bag. If a bag is labeled as negative, all instances within it are considered negative; conversely, if a bag is labeled as positive, at least one instance within it is positive. Multi-instance learning leverages neural networks to extract features from each instance and aggregates them into a feature representation of the bag. Subsequently, the classifier is trained at the bag level, enabling direct slide-level classification without the need for doctors to label patches with fine granularity.

However, current studies primarily focus on MIL-based WSI analysis at a single magnification level, while pathologists often switch between multiple magnifications to perform comprehensive diagnoses. Neglecting the multiplex information may lead to an incomplete understanding and interpretation of pathological patterns, thereby limiting the effectiveness of deep learning algorithms in capturing the full complexity of these images. Embracing the varied information present in different magnifications is essential to enhance the diagnostic accuracy and enable deep-learning models to encompass the richness of information contained within WSIs.

## 3 Materials and methods

### 3.1 Study design and workflow

**Figure 1:**
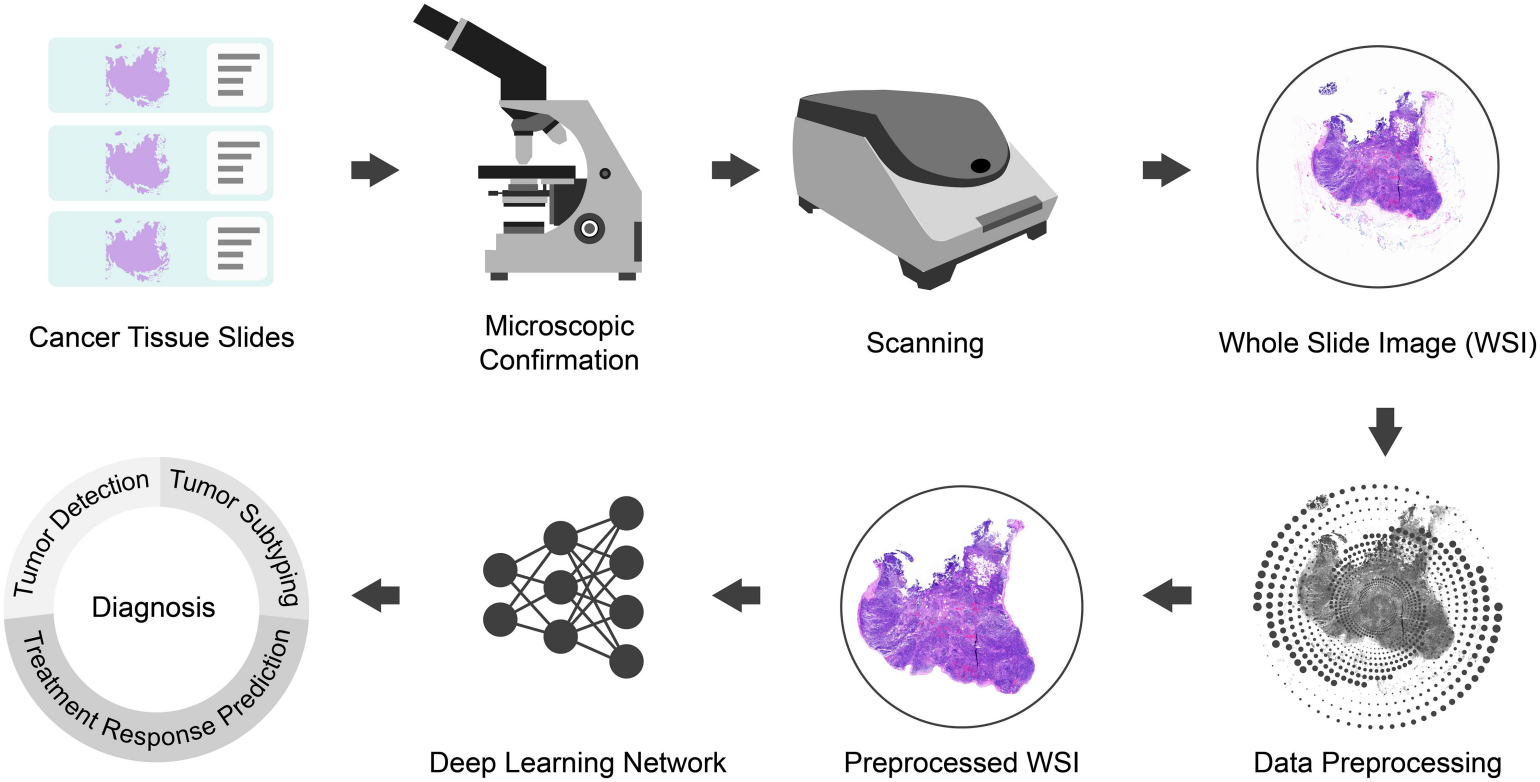
Pipeline of the whole study.

The present study focuses on advancing WSI processing through the integration of deep learning techniques. As illustrated in **Figure 1**, our methodology commences with the expertise of skilled pathologists, who meticulously prepare film and microscope reprints of tumor tissue sections. Subsequently, high-quality sections with clear labels are carefully chosen for digital scanning, yielding comprehensive WSI datasets. The utilization of WSIs is essential as it allows for a holistic view of the tissue, enabling a more comprehensive and accurate analysis.

To optimize the input data for deep learning analysis, the acquired WSIs undergo preprocessing (refer to Section 3.2). This step involves WSI patching, data normalization, and data augmentation, among other techniques, ensuring standardized and consistent data for subsequent analysis.

The main focus of our study involves the development and implementation of a sophisticated deep learning network, referred to as MAMILNet (detailed in section 3.2.3). By incorporating attention mechanisms, multi-instance learning, and multi-scale ensemble strategies, MAMILNet is strategically designed to effectively address the complexities associated with multiple prediction tasks across various cancer types. To comprehensively evaluate MAMILNet’s performance on these diverse prediction tasks, we utilized three data cohorts from four different centers, encompassing 1711 cases and WSIs. The predictive tasks for different cancer types include: automatic recognition of sentinel lymph node cancer in breast cancer, automatic typing of lung adenocarcinoma and lung squamous cell carcinoma, and evaluation of drug resistance in high-grade serous ovarian cancer.

### 3.2 Data collection and preprocessing

In this research, we conducted a comprehensive evaluation of MAMILNet’s performance on various prediction tasks for different cancer types using a total of 1711 cases and WSIs from three data cohorts across four centers. The predicted cancer types and tasks included the automatic recognition of sentinel lymph node cancer in breast cancer, automatic typing of lung adenocarcinoma and lung squamous cell carcinoma, and drug-resistance diagnosis of high-grade serous ovarian cancer. For details on the collection of relevant data queues, please refer to section 3.2.1, and for information on the pre-processing of data queues, see section 3.2.2.

#### 3.2.1 Data collection

##### 3.2.1.1 Breast cancer cohort

For the breast cancer cohort, we utilized the Camelyon 16 Dataset Bejnordi et al. (12), which is a prominent public benchmark in computational pathology, specifically focusing on sentinel lymph nodes. This dataset consists of a total of 399 whole-slide images (WSIs) collected from the Radboud University Medical Center in Nijmegen and the University Medical Center Utrecht in the Netherlands. Expert pathologists have annotated the tissue slides, labeling slides containing cancer as positive and those without cancer as negative. The raw data queue contains both slide-level weak labels and pixel-level labels for cancer regions. However, to adhere to the requirements of weakly supervised scenarios, we utilized only slide-level weak labels for training and testing purposes. This approach ensures the appropriate use of data while addressing the challenges posed by weak supervision in the context of this research.

##### 3.2.1.2 Lung cancer cohort

The TCGA lung cancer dataset comprises a total of 1050 H&E stained WSIs from the public Cancer Genome Atlas (TCGA) data portal. This dataset includes two subtypes of lung cancer: Lung Adenocarcinoma and Lung Squamous Cell Carcinoma. Expert pathologists have carefully annotated the WSIs, providing slide-level labels to indicate whether each WSI corresponds to Lung Adenocarcinoma (negative) or Lung Squamous Cell Carcinoma (positive). The meticulous annotations by expert pathologists ensure the accuracy and reliability of the dataset for further analysis and research in the field of lung cancer.

##### 3.2.1.3 Ovarian cancer cohort

The Ovarian Cancer Cohort comprises a total of 262 cases and WSIs from two centers: 228 patients from Yunnan Cancer Hospital, China, and 34 patients from Yunnan First People’s Hospital, China. After obtaining institutional review board approval, we retrospectively selected patients who received standardized treatment for ovarian cancer at Yunnan Cancer Hospital and Yunnan First People’s Hospital between 2015 and 2022.

Inclusion criteria for patient selection were as follows: (1) confirmation of high-grade serous ovarian cancer through operation and pathology; (2) treatment modalities including primary tumor cell reduction plus first-line platinum drug chemotherapy, or neoadjuvant chemotherapy plus tumor cell reduction plus first-line platinum drug chemotherapy; (3) availability of at least one pathological H&E-stained slide with focal lesions for each patient; (4) at least 6 months of available follow-up records after chemotherapy. Exclusion criteria included: (1) history of other malignant tumors, pelvic surgery, or platinum chemotherapy; (2) poor quality of tissue slides (Cases with poor slide quality, such as broken cap fragments or stains on the surface, insufficient tumor tissue, or tissue folding, were excluded.); (3) maximum diameter of the lesion less than 1 cm; (4) incomplete or substandard chemotherapy regimen; (5) incomplete clinical and pathological data. To clarify, at Yunnan Cancer Hospital, out of 270 patients considered, 42 were excluded based on the predefined criteria, resulting in 228 patients being included in the study. Similarly, at Yunnan First People’s Hospital, from an initial pool of 48 patients, 14 did not meet the inclusion criteria, leaving 34 patients to be enrolled in the study.

We defined platinum resistance as disease progression or recurrence within 6 months after the end of chemotherapy, and platinum sensitivity if there was no disease progression or recurrence within this timeframe. Tumor recurrence was determined based on histopathology or the presence of two of the following manifestations: sustained elevation of CA125, pleural effusion or ascites, physical examination finding a lump, imaging findings of a mass, or unexplained intestinal obstruction. For each patient, two professional pathologists Qinqing Wang and Qingyin Meng evaluated the slides, selecting 1-3 representative primary lesion slides. The images were then digitized through an off-field 20-magnification scan (0.48 *µ*m/pixel) using a portable scanner (Ocus, Grundium, Finland).

#### 3.2.2 Data preprocessing and partitioning

In this research, we employed the Python language (Version 3.7) and utilized the Openslide library (Version 3.4.1) to export all data queues at three magnifications: 20x, 10x, and 5x. We saved the resulting image sets separately, dividing them into non-overlapping 224×224 small image blocks. To ensure data quality, image blocks with an entropy of less than 5 were excluded, as they are likely to represent the background. For each resolution set, we performed image normalization using the mean and variance of all slices within the corresponding set. Data augmentation techniques, including random flipping, rotation, color transformation, and random cropping, were applied to enhance the dataset’s diversity. To achieve this, we utilized Python (Version 3.7) with libraries such as Pillow (Version 8.4.0), OpenCV (Version 4.1.0), and the PyTorch deep learning framework (Version 1.7.1). By employing these procedures and tools, we ensured that our dataset was prepared with standardized resolution and enhanced with data augmentation, setting a solid foundation for robust and reliable deep-learning model training and evaluation.

In the Breast cancer cohort, we conducted a random division to create a training set consisting of 240 cases and slides, and a test set containing 129 cases and slides. For the Lung Cancer Cohort, we applied a random division resulting in a training set comprising 840 cases and slides, and a test set comprising 210 cases and slides. Similarly, for the Ovarian Cancer Cohort, we randomly divided it into a training set with 183 cases and slides, and a test set with 79 cases and slides. Importantly, each of the training sets also includes validation sets.

#### 3.2.3 Multi-scale attentional multi-instance learning network

**Figure 2:**
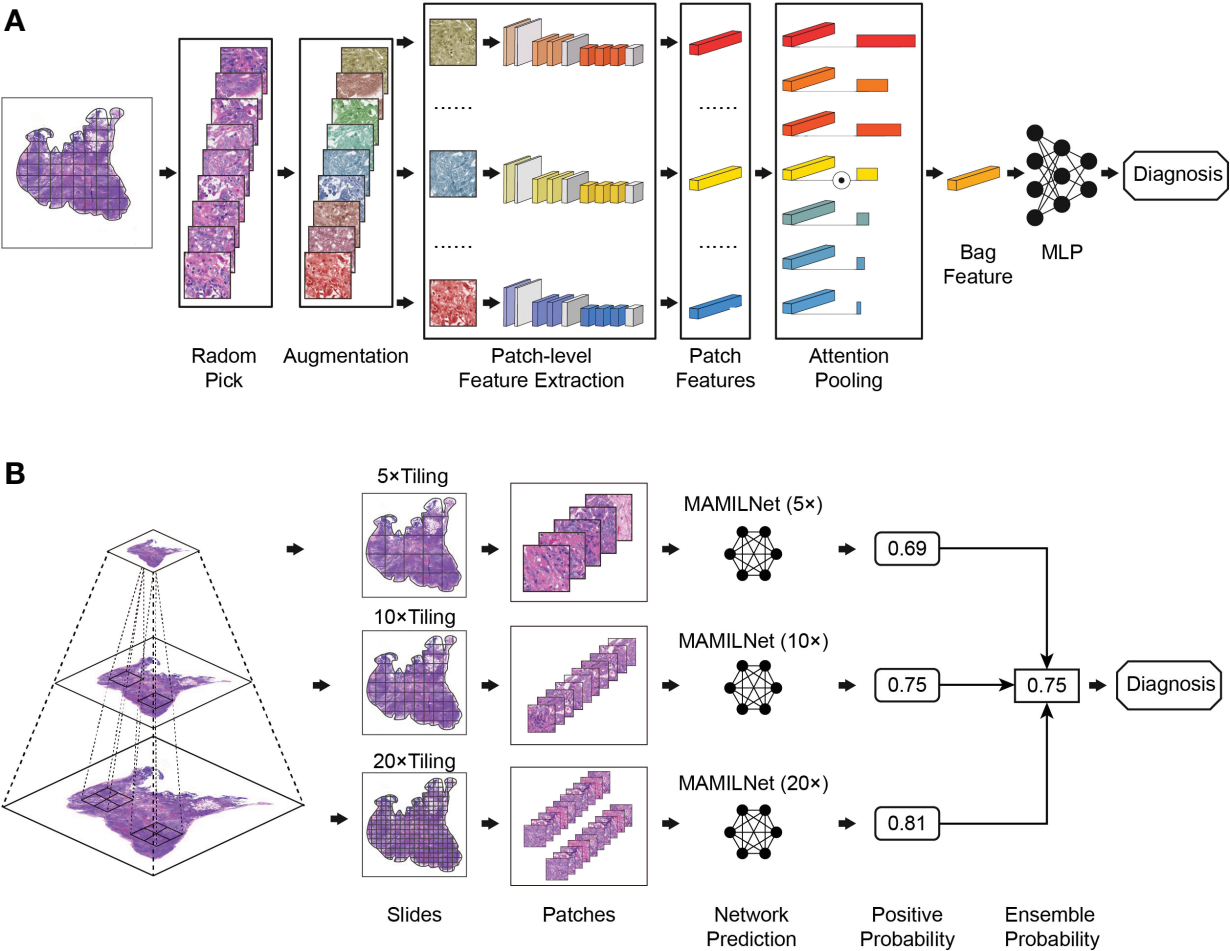
**(A)** Training process of the MAMILNet; **(B)** Inference process of the MAMILNet.

We present MAMILNet, a multi-instance deep convolutional neural network architecture incorporating a multi-scale attentional mechanism, designed to handle multiple prediction tasks for various cancers. The network’s training process is illustrated in **Figure 2A**. During training, we create separate models for the 20x, 10x, and 5x image sets, as depicted in **Figure 2A**. Each set of patches from the same WSI constitutes a bag. Before each iteration, we apply random data augmentation techniques to each patch in the bag, including random noise, rotation, clipping, and color transformation. Next, we utilize a pre-trained ResNet He et al. (27) network as the primary feature extractor to obtain the features of each patch within the bag. Subsequently, an attention module is employed, where a learnable attention weight is assigned to the features of each image block. This attention-pooling process aggregates the features within the bag to obtain the bag-level features. Finally, a bag-level Multilayer Perceptron (MLP) serves as the bag classifier, directly predicting the negative and positive risks of the WSI. The cross-entropy loss, calculated against the true labels, serves as the loss function during training, and stochastic gradient descent drives the parameter updates in the network.

The attention module consists of two main steps. First, a linear fully connected layer reduces the dimension of each feature vector to 128, followed by the application of the pixel-level hyperbolic tangent function (tanh()). This non-linear output is then scaled to be between -1 and 1. The resulting values are multiplied with another linear layer to calculate the attention weight of each image block using the softmax function. In the second step, we use the feature matrix of the image block and the attention weight matrix to obtain the bag-level features. This step involves element-wise multiplication of the feature matrix with the attention weight matrix, effectively emphasizing the important regions within the bag based on their calculated attention weights. The resulting features represent a compact representation of the bag, capturing the salient information required for accurate bag-level predictions.

The inference process of the network is depicted in **Figure 2B**. During the testing phase, we propose a multi-scale integration strategy inspired by pathologists who often zoom in and out of slices for diagnosis. This strategy allows us to make the final prediction for the test cohort. Specifically, we employ the 20x, 10x, and 5x models obtained during the training process to calculate the predicted risk probability for each WSI at each magnification level. Next, we use the mean-pooling method to aggregate the prediction probabilities from the three magnification levels. This aggregation process yields the final prediction risk probability for each WSI.

We utilized the PyTorch deep learning framework (Version 1.7.1) in Python (Version 3.7) to perform all training and testing processes of the network. The Adam optimizer was employed to train the model, utilizing the cross-entropy loss as the loss function. The total number of training iterations was set to 500, with a learning rate of 1e-4. We applied a weight decay factor of 1e-5, and the batch size was set to 64. For computational resources, we conducted the training using the 11th Gen Intel(R) Core(TM) i7-11700K CPU in combination with the Nvidia 3090 GPU. These hardware configurations ensure efficient and high-performance processing during the training and testing phases of our deep-learning model.

#### 3.2.4 Statistical analysis

In our specific experiments, we employed the cross-validation method to train the model and select the best-performing model for final internal verification and independent testing. During this process, the verification set was utilized to identify the model with the optimal performance, while the independent test set remained unseen during both the training and verification stages, ensuring a fair evaluation of the model’s performance. Specifically, for each dataset, we first divide it into a training set (including a validation set) and an independent test set at a ratio close to 4:1, where the independent test set remains unseen during the training and validation process. The details of the division can be found in Section 3.2.2 Data Preprocessing and Partitioning. For the training set, we employed a 5-fold cross-validation method. This technique divides the training dataset into five parts, using four parts for training and one part for validation in each iteration. This process ensures that each data point is used for both training and validation once, thus obtaining a more reliable estimate of model performance. Then we select the best model and parameters from the cross-validation to test on the independent test set and report the results of the independent test set as the final outcome. This also better simulates the prediction scenario for more new unseen clinical data in the future.

To assess the model’s performance, we employed several metrics, including the area under the ROC curve (AUC), Accuracy, False Positive Rate (FPR), and False Negative Rate (FNR). These metrics were reported along with 95% confidence intervals (CI) to provide a comprehensive understanding of the model’s effectiveness. All metric calculations and statistical analyses were conducted using the scikit-learn package (Version 1.3.0) within the Python (Version 3.7) environment. The scikit-learn package offers robust and reliable tools for evaluating machine learning models, ensuring the accuracy and consistency of our model assessments.

## 4 Results

### 4.1 Prediction results of sentinel lymph node tumor detection in breast cancer

In the task of tumor diagnosis of sentinel lymph nodes of breast cancer, as shown in **Table 1**, our innovative MAMILNet demonstrated remarkable success, achieving an impressive AUC of 0.8872 (95%CI 0.86-0.90) on the independent test set. Moreover, our model exhibited high accuracy (0.8760, 95%CI 0.85-0.89) and demonstrated low false positive rate (FPR=0.1406, 95%CI 0.16-0.12) and false negative rate (FNR=0.1077, 95%CI 0.08-0.12) performances. These compelling results underscore the efficacy of MAMILNet in accurately diagnosing tumors based on H&E-stained WSIs, and hold significant promise for enhancing breast cancer diagnostics and patient outcomes.

**Table 1:** Prediction results on the independent test set of sentinel lymph node tumor detection in breast cancer.

Deep-learning Model	AUC	Accuracy	FNR	FPR
5× only MAMILNet	0.7684	0.7520	0.2258	0.2698
10× only MAMILNet	0.8379	0.8217	0.1384	0.2187
20× only MAMILNet	0.8653	0.8450	0.1538	0.1562
MILRNN Campanella et al. (14)	0.8178	0.8062	0.1428	0.2542
CLAM Lu et al. (28)	0.8762	0.8527	0.1142	0.2000
**MAMILNet (ours)**	**0.8872**	**0.8760**	**0.1077**	**0.1406**

Bold values refer to the best results.

Moreover, by comparing our multi-scale model with MAMILNet variants and advanced competitors, we observed further improvements through our proposed multi-scale integrated prediction strategy, validating its effectiveness in enhancing model performance. These findings signify significant strides in the field of deep learning-based tumor diagnosis, propelling advancements in early detection and precision medicine for breast cancer patients.

**Figure 3:**
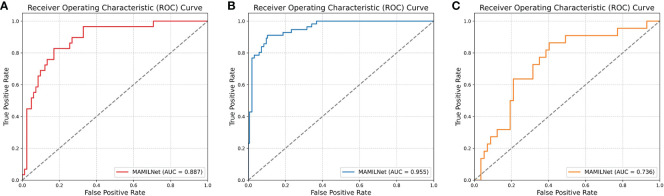
**(A)** The ROC curve of MAMILNet on the breast cancer sentinel lymph node tumor detection task (independent test set). **(B)** The ROC curve on lung cancer tumor typing task (independent test set). **(C)** The ROC curve on the ovarian cancer treatment resistance prediction task (independent test set).

The ROC curve of MAMILNet on the breast cancer sentinel lymph node tumor detection task on the independent test set is shown in **Figure 3A**.

### 4.2 Prediction results of automatic subtyping of lung adenocarcinoma and lung squamous cell carcinoma

In the domain of WSI processing for lung cancer typing diagnosis, as can be seen from **Table 2**, our MAMILNet demonstrated outstanding performance. The achieved AUC of 0.9551 (95%CI 0.94-0.96) on the independent test set, coupled with accuracy of 0.9095 (95%CI 0.89-0.91), FPR of 0.0961 (95%CI 0.110.07), and FNR of 0.0857 (95%CI 0.09-0.07), affirms the model’s accurate and efficient classification of lung tumor types based on H&E-stained WSIs. Additionally, a comparative analysis with other single-scale variants and competitors underscores the superiority of our innovative multi-scale strategy, further validating its efficacy in enhancing classification accuracy and diagnostic performance. These findings represent a significant advancement in the field of deep learning-based lung cancer typing, offering promising avenues for improving patient care and treatment outcomes.

**Table 2:** Prediction results on the independent test set of automatic subtyping of lung adenocarcinoma and lung squamous cell carcinoma.

Deep-learning Model	AUC	Accuracy	FNR	FPR
5× only MAMILNet	0.9269	0.8619	0.1333	0.1429
10× only MAMILNet	0.9324	0.8904	0.0952	0.1250
20× only MAMILNet	0.9488	0.9000	0.0857	0.1153
MILRNN Campanella et al. (14)	0.9236	0.8667	0.1111	0.1733
CLAM Lu et al. (28)	0.9411	0.8857	0.0714	0.2000
**MAMILNet (ours)**	**0.9551**	**0.9095**	**0.0857**	**0.0961**

Bold values refer to the best results.

The ROC curve on lung cancer tumor typing task on the independent test set is shown in **Figure 3B**.

### 4.3 Prediction results of drug resistance in high-grade serous ovarian cancer

We explored a novel and challenging clinical task—predicting patients’ drug therapy response using the Ovarian Cancer Cohort. This task, which surpasses the interpretability of H&E-stained slides even for medical experts, represents a pressing problem in contemporary medical research. As can be seen from **Table 3**, our MAMILNet demonstrated promising results in this endeavor, achieving an AUC of 0.7358 (95%CI 0.74-0.72) on the independent test set, along with accuracy of 0.7341 (95%CI 0.72-0.74), FPR of 0.2982 (95%CI 0.30-0.28), and FNR of 0.1818 (95%CI 0.17-0.19). These performance indicators signify the potential of our MAMILNet to make significant advancements in drug response therapy prediction, ushering in a new era of personalized medicine and improved patient outcomes in ovarian cancer management.

**Table 3:** Prediction results on the independent test set of drug resistance in high-grade serous ovarian cancer.

Deep-learning Model	AUC	Accuracy	FNR	FPR
5× only MAMILNet	0.6741	0.6582	0.3636	0.3333
10× only MAMILNet	0.6968	0.6835	0.2727	0.3333
20× only MAMILNet	0.7126	0.7088	0.2727	0.2982
MILRNN Campanella et al. (14)	0.6537	0.6329	0.3333	0.4118
CLAM Lu et al. (28)	0.6884	0.6709	0.2727	0.4000
MAMILNet (ours)	0.7358	0.7341	0.1818	0.2982

The ROC curve on the ovarian cancer treatment resistance prediction task on the independent test set is shown in **Figure 3C**.

## 5 Discussion and conclusions

This research introduces MAMILNet, a novel multi-scale attentional multi-instance learning framework, which achieves remarkable performance in critical tasks like breast cancer tumor detection, lung cancer subtype diagnosis, and ovarian cancer drug resistance prediction, even with weak slide-level labeling.

MAMILNet’s innovative design and capabilities hold significant promise for advancing medical image analysis, improving diagnostic accuracy, and guiding cancer treatment decisions.

MAMILNet demonstrates its robust performance through three key components. Firstly, it effectively employs attention mechanisms to adaptively learn critical visual features associated with various cancer species and tasks. By assigning higher attention weights to clinically relevant visual features and lower weights to unrelated background and noise, MAMILNet acts as a dynamic filter, enhancing its learning ability for different tasks. Consequently, the network exhibits exceptional generalization across multiple cancer types and prediction tasks. Secondly, MAMILNet adopts a feature aggregation-based multi-instance learning architecture, enabling remarkable performance even with slide-level weak annotation. It treats slides as “bags” and their cut patches as “instances,” employing an efficient instance-level feature extractor to derive informative representations. An attention mechanism is then used to aggregate these instance features effectively into bag-level features. This approach culminates in a powerful bag classifier, enabling accurate slide-level classification. The combination of instance-level efficient feature representation, attention-based feature aggregation, and a robust bag-level classifier ensures MAMILNet’s efficiency. Lastly, inspired by pathologists’ “zoom in and out” reading approach, MAMILNet introduces a multi-scale “consultation” training and prediction strategy. During training, it uses multiple magnifications to fully model and learn pathological features. During testing, a probabilistic set approach aggregates results from different scales, harnessing the advantages of diverse magnifications for optimal prediction performance, akin to a medical consultation process. This innovative strategy further enhances MAMILNet’s predictive capabilities.

Tumor detection and pathologic subtyping are vital in WSI analysis through deep learning. The deep learning model offers faster and more detailed diagnostic references directly from H&E-stained slides, significantly reducing examination time. Moreover, its highly generalized nature facilitates diagnosis in regions with limited medical resources. This study demonstrates satisfactory performance in both tasks, paving the way for the widespread application of deep-learning models in this domain. It is also among the pioneering efforts to explore the direct prediction of drug response therapeutic efficacy from WSI using deep learning. Assessing a patient’s response to drug therapy is critical for treatment decisions and prognosis. However, determining drug resistance from H&E-stained slides is challenging, even for experienced physicians. Studies Vamathevan et al. (29) Ballester et al. (30) Farahmand et al. (31) suggest that a patient’s sensitivity to specific drug therapy may be evident in H&E-stained sections, presenting opportunities for deep-learning model applications. The deep-learning model effectively captures pathological patterns related to drug treatment responses in a data-driven manner, enabling accurate predictions. The research conducted a preliminary trial on High-grade Serous Ovarian Cancer, yielding promising results. These findings support the future prediction of treatment responses using deep learning across various cancer types with different drugs. This breakthrough holds significant potential for advancing personalized medicine and enhancing patient outcomes.

The study has several limitations that require careful consideration. Firstly, it adopts a retrospective analysis approach, which may inherently constrain the diversity and representativeness of the data. Future investigations aim to build a larger and more comprehensive dataset to enhance the model’s robustness. Secondly, for drug resistance prediction tasks, systematic pathologic patterns have not been identified. The deep-learning model relies on data-driven iteration and training, making it challenging to understand the underlying pathological basis of its judgments. Although the designed attention mechanism can highlight patches of high and low risk, further experiments are needed to systematically generalize authoritative pathological patterns. While this study provides a preliminary glimpse into the potential of utilizing deep-learning models for drug therapy response prediction in ovarian cancer, full generalization of this knowledge requires more extensive trials and investigation. Thirdly, despite conducting trials involving three cancer species from four centers, the validation across multiple centers remains insufficient for each task. Limited multi-center training and verification constrain the generalization and robustness of deep learning networks. To address this, future endeavors will focus on collecting more extensive data to facilitate large-scale, multi-center training and validation, ensuring more comprehensive and reliable results.

In our study, we investigated the predictors of therapeutic outcomes in ovarian cancer, acknowledging that these are influenced by a range of factors beyond tumor morphology, such as pathological stage, resection margins, patient performance status, and comorbidities. We developed a deep learning model, using pathological slides as the sole input, to predict drug resistance, exploring the potential of pathological sections as an independent biomarker. While integrating clinical and pathological data could improve predictive accuracy, our initial focus on pathological slides due to scope and time constraints represents a deliberate first step towards a comprehensive research strategy. Plans to include clinical data in future analyses acknowledge the opportunity to enhance drug resistance predictions. We analyzed additional clinical data, including Federation International of Gynecology and Obstetrics (FIGO) stage, age, and BMI, through logistic regression to assess their relationship with platinum resistance. The p-values for these factors (0.2052, 0.9191, and 0.3393, respectively) suggest they are poor predictors of platinum resistance, as evidenced by AUC values of 0.58, 0.51, and 0.54 in independent tests. Conversely, our deep learning analysis of pathological images with MAMILNet demonstrated higher predictive accuracy for treatment response, achieving an AUC of 0.7358, with significant accuracy, FPR, and FNR rates. We aim to extend our research to include broader clinical parameters, enhancing prediction accuracy and understanding of therapeutic outcome determinants in ovarian cancer. This multidimensional approach promises to refine our predictive models and contribute valuable insights into the complex dynamics of cancer treatment response.

In summary, deep-learning-based WSI analysis has emerged as a crucial approach for cancer diagnosis. This study introduces a novel multi-scale attentional multi-instance network architecture (MAMILNet), presenting a fresh perspective and method for WSI analysis using deep learning. Notably, MAMILNet demonstrates promising results in breast cancer tumor detection, lung cancer tumor typing, and ovarian cancer drug resistance prediction tasks. These achievements offer valuable insights for the wider application of deep-learning models in these areas and inspire new avenues for utilizing deep learning in diverse cancer types and diagnostic tasks. MAMILNet’s performance signifies its potential as a powerful tool for enhancing cancer diagnosis and treatment in clinical settings.

## Data availability statement

The original contributions presented in the study are included in the article/supplementary material. Further inquiries can be directed to the corresponding author.

## Ethics statement

The studies involving humans were approved by the First People’s Hospital of Yunnan Province. The studies were conducted in accordance with the local legislation and institutional requirements. The human samples used in this study were acquired from primarily isolated as part of your previous study for which ethical approval was obtained. Written informed consent for participation was not required from the participants or the participants’ legal guardians/next of kin in accordance with the national legislation and institutional requirements. The manuscript presents research on animals that do not require ethical approval for their study. Written informed consent was obtained from the individual(s) for the publication of any potentially identifiable images or data included in this article.

## Author contributions

KM: Conceptualization, Data curation, Investigation, Project administration, Resources, Supervision, Writing – review & editing. QW: Conceptualization, Data curation, Formal analysis, Investigation, Methodology, Validation, Writing – original draft. QB: Conceptualization, Data curation, Formal analysis, Investigation, Methodology, Resources, Validation, Writing – original draft. LQ: Conceptualization, Methodology, Software. YD: Data curation, Investigation, Resources. XW: Data curation, Investigation, Resources. YZ: Data curation, Investigation, Resources. CL: Data curation, Investigation, Resources. QM: Conceptualization, Resources, Supervision, Writing – review & editing.
